# Colony size is linked to paternity frequency and paternity skew in yellowjacket wasps and hornets

**DOI:** 10.1186/s12862-014-0277-x

**Published:** 2014-12-30

**Authors:** Kevin J Loope, Chun Chien, Michael Juhl

**Affiliations:** Department of Neurobiology and Behavior, Cornell University, Ithaca, NY USA; Bee Man Exterminators LLC, Olympia, WA USA

**Keywords:** Social insects, Polyandry, Multiple paternity, Paternity skew, Vespula, Dolichovespula, Vespa, Social evolution

## Abstract

**Background:**

The puzzle of the selective benefits of multiple mating and multiple paternity in social insects has been a major focus of research in evolutionary biology. We examine paternity in a clade of social insects, the vespine wasps (the yellowjackets and hornets), which contains species with high multiple paternity as well as species with single paternity. This group is particularly useful for comparative analyses given the wide interspecific variation in paternity traits despite similar sociobiology and ecology of the species in the genera *Vespula*, *Dolichovespula* and *Vespa*. We describe the paternity of 5 species of yellowjackets (*Vespula* spp.) and we perform a phylogenetically controlled comparative analysis of relatedness, paternity frequency, paternity skew, colony size, and nest site across 22 vespine taxa.

**Results:**

We found moderate multiple paternity in four small-colony *Vespula rufa-*group species (effective paternity 1.5 – 2.1), and higher multiple paternity in the large-colony *Vespula flavopilosa* (effective paternity ~3.1). Our comparative analysis shows that colony size, but not nest site, predicts average intracolony relatedness. Underlying this pattern, we found that greater colony size is associated with both higher paternity frequency and reduced paternity skew.

**Conclusions:**

Our results support hypotheses focusing on the enhancement of genetic diversity in species with large colonies, and run counter to the hypothesis that multiple paternity is adaptively maintained due to sperm limitation associated with large colonies. We confirm the patterns observed in taxonomically widespread analyses by comparing closely related species of wasps with similar ecology, behavior and social organization. The vespine wasps may be a useful group for experimental investigation of the benefits of multiple paternity in the future.

**Electronic supplementary material:**

The online version of this article (doi:10.1186/s12862-014-0277-x) contains supplementary material, which is available to authorized users.

## Background

The mating frequency of social insect queens is a central factor shaping the evolution of social behavior within colonies. Polyandry, and the genetic diversity created by multiple paternity, is the foundation of many evolutionary conflicts within insect societies [1]. It also has important consequences for sexual selection, sperm competition, and the evolution of male reproductive strategies [2,3]. But the evolution of multiple mating and multiple paternity itself is an evolutionary puzzle. It has arisen several times in the social Hymenoptera, including in a handful of ant genera, the honey bees (*Apis* spp.), and some vespine wasps (e.g. *Vespula* spp.) [4]. Multiple paternity presents automatic costs of increased exposure to sexually transmitted disease, greater predation risk while mating, and greater potential conflict among colony members due to lower relatedness [5]. So, given these costs, what are the benefits that underlie the adaptive maintenance of multiple paternity?

**Figure 1:**
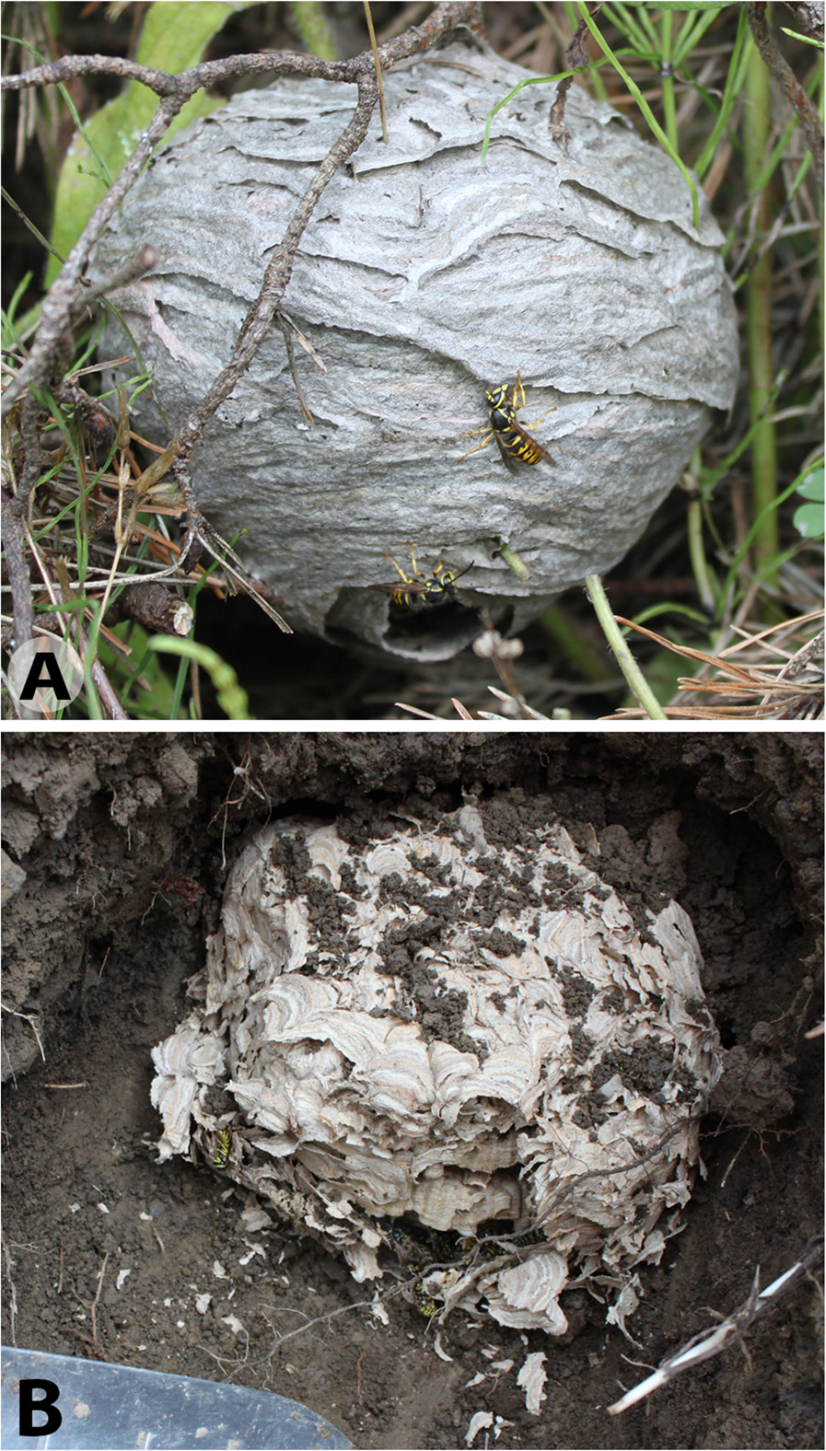
**Aerial and subterranean yellowjacket nests. A**. Young colony of *Dolichovespula arenaria*, a typical aerial-nesting yellowjacket. **B**. Excavated, subterranean nest of an anesthetized colony of *Vespula flavopilosa.*

Many hypotheses have been proposed to explain the fitness benefits of multiple mating and multiple paternity in the eusocial Hymenoptera (reviewed in [5-8]). The most successful suggest a colony-level benefit derived from the greater genetic diversity of colony members created when a queen uses sperm from multiple males. The pathogens and parasites hypothesis proposes that multiple paternity results in a colony with diverse genetic defenses against coevolving natural enemies, thus reducing intracolony disease transmission and increasing colony survival [9-12]. Alternatively, the division of labor hypothesis suggests that colonies with greater genetic diversity have greater genetically determined behavioral diversity or broader task performance thresholds, which allows for a more efficient division of labor or the exploitation of rare genetic specialists [6,13-16]. A third popular hypothesis not based on genetic diversity is that queens mate with multiple males to acquire a sufficient number of sperm [17]. In this paper we explore the evolution of multiple paternity, and the predictions of these hypotheses, by comparing species of vespine wasps, i.e., the yellowjackets and hornets (Figure 1). The vespine wasps share many features of their ecology and sociobiology but vary dramatically in paternity [18-20], making them a useful group for comparative studies of the evolution of multiple paternity and its consequences.

**Figure 2:**
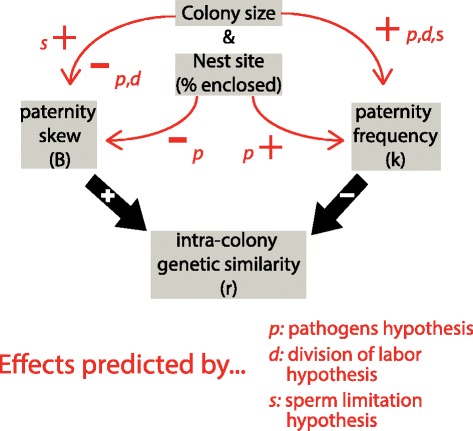
**Predicted effects of colony size and nest site on paternity traits.** Red arrows indicate the predicted positive (+) or negative (−) effects of increasing colony size and frequency of cavity nesting on paternity skew and paternity frequency. The predictions of the pathogens hypothesis and division of labor hypothesis stem from the effects of paternity traits on intracolony genetic similarity (black arrows).

The pathogens hypothesis, the division of labor hypothesis and the sperm limitation hypothesis all predict that colony size (i.e. the number of workers in a colony) will be positively associated with paternity frequency (i.e. the number of fathers represented in the workers of a given colony; Figure 2) [11,17,21]. A larger workforce inevitably results in increased traffic by returning foragers. If each foraging trip represents a possible entry into the nest of an externally encountered pathogen, then larger colonies will acquire forager-borne diseases and parasites sooner and more often [11]. This higher parasite pressure would cause larger colonies to benefit more from disease resistance conferred by multiple paternity. Species with large colonies also tend to have greater division of labor and specialization, and thus will benefit more from multiple paternity if the genetic diversity it brings enhances the division of labor [14,21]. Both of these hypotheses also predict more even sperm use (reduced paternity skew) for species with greater colony size because reducing skew leads to reduced intracolony genetic similarity (Figure 2; [22]). Finally, queens who create large colonies may require more sperm than is provided by her first mate, and would thus be selected to mate with more males, increasing paternity frequency [17]. In the only study of male sperm quantity in vespine wasps, adult males were found to contain over 2 million sperm, approximately 100 times the average number found in the sperm storage organs of spring queens [23], suggesting sperm may not be limiting. However, it remains theoretically possible that males benefit from incompletely inseminating queens due to sexual conflict [24]. Unlike the genetic-diversity based hypotheses, the sperm limitation hypothesis predicts that paternity skew should increase with colony size, because queens in large colonies should be selected to use all available sperm and males likely range widely in the amount of sperm they provide (Figure 2; [22]).

Comparative analyses have shown that across taxonomically broad sets of species, there is a positive relationship between paternity frequency and colony size [11,17,22], as well as a negative relationship between paternity skew and paternity frequency, in ants, bees, and wasps [22]. As the selective forces that drive the evolution of polyandry may differ across the social insects [5], our aim in this work is to examine these predictions in a clade possessing a wide range of paternities, with species that differ little in social complexity and other traits that might confound the explanation of multiple paternity.

For the vespine wasps, we extend the logic of the pathogens hypothesis to propose another factor–nest site–that may be involved in the evolution of multiple mating and multiple paternity. Vespine wasps all construct similar nests of multiple combs surrounded by insulating layers of paper envelope (Figure 1). Some species build nests hidden in cavities, typically excavated rodent burrows, rotten logs or tree holes. Other species construct exposed, aerial nests in shrubs or suspended from tree branches. It seems possible that these nest sites, and their microenvironments, expose colonies to different types and quantities of pathogens, as has been suggested for canopy- and soil-dwelling ants [25]. Cavity-nesting and ground-nesting species may experience greater exposure to fungal and other microbial pathogens due to the increased proximity to damp soil and rotting wood, which could in turn favor multiple paternity. A casual examination of the vespine species included in the most extensive comparative analysis of colony size and paternity [22] suggests an association between colony size, nest site, and paternity. The large-colony, *vulgaris*-group *Vespula* species are subterranean nesting and have high paternity, while the small-colony *Dolichovespula* species are aerial nesting and have a low paternity. Because nest site and colony size are tightly associated in this data set, it is unclear whether it is colony size or nest site (or both) that distinguishes high-paternity species from low-paternity species.

Here we assume that colony size and nest site are determined by ecological factors [21], such as climate, prey type and availability, nest site availability and predator type and abundance. These hypotheses predict that selection then modifies mating and paternity traits to reflect the ecologically determined colony size and nest site. In short, these hypotheses predict that colony size and nest site cause changes in mating behavior and paternity, rather than vice versa (Figure 2).

In this study we describe the paternity of five species of North American *Vespula* wasps. Four of these species are members of the enigmatic *Vespula rufa* group, which have small colonies and subterranean nests [26,27]. These features make this clade attractive for testing the link between nest site and paternity as these species break the correlation between colony size and nest site found in the species that have been previously studied. The fifth species, *Vespula flavopilosa*, has small colonies compared to other species in the *Vespula vulgaris* group [28], and may thus provide an interesting intermediate position in the comparison of colony size and mating frequency. It is also a facultative social parasite [28], a feature sometimes associated with reduced paternity [29,30]. We then perform a phylogenetically controlled comparative analysis, including both colony size and nest site, to examine the species-level traits associated with the evolution of intracolony genetic similarity (relatedness), paternity frequency and paternity skew in the Vespinae.

## Methods

### Colony collections

We collected workers from active, mature colonies located by responding to pest control calls (*V. consobrina, V. atropilosa* and *V. acadica* in Thurston, Co., WA), by nest searching (*V. flavopilosa*; Tompkins Co., NY), or by “wasp-lining” foragers back to their nests (*V. vidua*; Tompkins Co., NY). All collections adhered to state and federal regulations. Most colonies would have been destroyed as pests regardless of our collection, and none are species known to be endangered or threatened. Samples were collected between 2008–2013 and stored frozen (−20 deg C). We collected entire colonies of *V. consobrina*, *V. vidua* and *V. flavopilosa* to obtain colony size data; a sample of worker *V. acadica* and *V. atropilosa* were collected from the nest entrance with a vacuum. All *V. consobrina* and some *V. vidua* colonies were collected during the day using a battery-powered vacuum. The collector waited at least 30 minutes to collect returning foragers, and for *V. vidua*, we returned hours later to collect the last remaining foragers and escapees. All colonies of *V. flavopilosa* and some colonies of *V. vidua* were anesthetized overnight with CO_2_ and excavated in the morning. For colonies of *V. consobrina*, *V. vidua* and *V. flavopilosa*, we counted all adult workers and determined the presence or absence of the mother queen.

### Genetic analysis

We extracted DNA from approximately twenty workers or gynes per colony, as well as the mother queen when present, by placing a single antenna or leg in 100 μL of 10% Chelex solution (Chelex 100, 100–200 mesh, Bio-Rad), then incubating for 20 minutes at 95°C. We then refrigerated or froze the supernatant before PCR. Variable loci were selected based on preliminary screening of published loci [31-33]. We used dye-labeled primers (Applied Biosystems) in combination with a 3-primer labeling method [34] to perform multiplex PCR with 4–6 primers, depending on the species (Additional file 1: Table S1). Each 10 μL PCR reaction included 1ul extracted DNA, 5 μL Qiagen master mix (Qiagen Type-It Microsatellite Kit, Qiagen Inc.), 0.2 μL of each reverse primer, 0.2 μL (dye-labeled) or 0.1 μL (3-primer labeled) of each forward primer, 0.15 μL FAM-labeled 3-primer tag for each 3-primer-labeled primer pair, and water to total 10 μL. PCR reaction conditions were 95°C for 15 minutes, 35 cycles of 95°C for 30 seconds, 50°C for 90 seconds, 72°C for 60 seconds, followed by 60°C for 30 minutes. Fragment analysis was performed on an ABI-3730 × l sequencer using 0.5 μL PCR product combined with 15 μL HiDi Formamide and 0.15 μL LIZ 500 internal size standard (Applied Biosystems). Allele sizes were called using GeneMarker (SoftGenetics LLC) and checked twice by eye.

### Estimating paternity

We used Colony2 v2.0.4.1 [35] to find the maximum likelihood configuration of paternity assignments for all genotyped workers. Workers that failed to amplify at more than two loci were excluded from the analysis. In the few cases where Colony2 assigned a worker to a matriline from a different colony, the anomalous worker genotype was checked against the genotype of the queen from that colony. If she shared an allele with the queen at all loci, a maternal sibship constraint was entered into Colony2 containing all workers in that colony that did not differ from the queen for both alleles at any locus. In all cases, the subsequent run of the likelihood analysis assigned the worker to an additional patriline from that colony. In the few cases where anomalous workers were inconsistent with being a daughter of the queen, these workers were removed from the dataset and the analysis was run again. From the paternity assignments, we calculated each colony’s observed paternity frequency (*k*), an uncorrected estimate of effective paternity: ${k}_e=1/{\displaystyle {\sum}_{i=1}^k{p}_i^2}$ where *p* is the proportion of offspring in the sample fathered by male *i* [7,36,37], and a corrected estimate of effective paternity (*k*_*e3*_), which adjusts for sample size [37]. Mean intracolony relatedness was calculated from effective paternity using the formula $\frac{1}{4}+\frac{1}{2{k}_{e3}}$. We calculated paternity skew using the B index [38]. Additional file 1: Table S1 reports allelic diversity and expected heterozygosity calculated from allele frequencies determined by Colony2.

### Non-detection and non-sampling error

Two types of error—non-detection and non-sampling of fathers—could lead to an inaccurate estimation of effective paternity [7]. Non-detection error, when two males have the same multilocus genotype, was estimated using the formula found in ref [39]. For all five species studied, the non-detection error was <0.0025 (Table 1), suggesting that such errors did not bias our estimate of effective paternity. However, this calculation assumes maternal and paternal genotypes are known [7,40]. When examining the maternal genotype assignments following maximum likelihood analysis in Colony2 for colonies without a genotyped queen, queen genotypes were occasionally ambiguous (i.e., assigned a genotype with probability <0.9). There were 7 ambiguous single-locus genotypes for queens of *V. acadica*, 3 for *V. vidua,* and 2 for *V. atropilosa*. This never occurred at more than one locus per queen, so a simple and conservative estimate of the upper bound of male non-detection error is to remove the single most variable of the problematic loci from the non-detection calculation for each of these species. Even with this adjustment, error rates were low enough to be confident that paternity estimates were not biased due to male non-detection error (Table 1).

**Table 1:** **Summary data from paternity analysis of five** ***Vespula*** **species**

	**Site**	**Year**	***n***_***c***_	***n***_***w***_	***k***	***k***_***e***_	***k***_***e3***_	***B***	**# W**	**NDE**
*V. acadica*	WA	2008-2013	10	19.9 (19, 20)	2.00 (1.42, 2.58)	1.47 (1.17, 1.98)	1.51 (1.18, 2.08)	0.044	-	4.7e-4^*a*^ (1.3e-3)^*b*^
*V. atropilosa*	WA	2008-2013	10	20.1 (19, 23)	2.40 (1.80, 3.00)	1.67 (1.37, 2.14)	1.72 (1.39, 2.26)	0.10	-	2.5e-3^*a*^ (1.3e-2)^*b*^
*V. consobrina*	WA	20082013	12	20.4 (18, 24)	2.83 (2.00, 3.66)	1.82 (1.43, 2.50)	1.89 (1.46, 2.68)	0.069	98 (52.0)	8.0e-6
*V. vidua*	NY	2012-2013	10	19.8 (18, 21)	3.00 (2.12, 3.88)	2.01 (1.62, 2.67)	2.12 (1.67, 2.90)	0.056	172 (103.3)	1.7e-5^*a*^ (4.1e-5)^*b*^
*V. flavopilosa*	NY	2012-2013	10	19.6 (18, 20)	3.80 (3.10, 4.50)	2.80 (2.34, 3.49)	3.07 (2.50, 3.97)	0.033	899 (718.2)	1.4e-3

Colony data used to generate these summary values are presented in Additional file 1: Table S2. Site: WA collection occurred in Thurston, Co., Washington. NY collection occurred in Tompkins Co., New York. *n*_*c*_: number of colonies analyzed. *n*_*w*_: arithmetic mean (range) of the number of female offspring genotyped per colony. *k*: arithmetic mean (95% CI) of the number of male mates detected. *k*_*e*_: harmonic mean (95% CI) of the uncorrected estimate of effective paternity. *k*_*e3*_: harmonic mean (95% CI) of effective paternity corrected for sample size [37]. B: arithmetic mean paternity skew using the *B* index [38]. # W: arithmetic mean (SD) number of workers collected in mature colonies. NDE: male non-detection error (see text). *a*. estimate for male non-detection error assuming all parental genotypes are known. *b.* upper estimate for male non-detection error, accounting for uncertain parental genotypes.

Non-sampling error occurs when a father is not detected because the sampled daughters do not include his offspring. We used the formula presented in [41,42] to estimate the expected number of patrilines per colony, given the observed number of patrilines and the number of individuals sampled, assuming no skew among patrilines. The species-level average expected paternity frequencies were virtually identical to the observed values (V*. acadica:* 2.00, *V. atropilosa*: 2.40, *V. vidua*: 3.02, *V. consobrina*: 2.85, *V. flavopilosa*: 3.84; compare to observed values in Table 1). Our estimates of effective paternity and thus relatedness are already corrected for non-sampling, since the calculation of k_e3_ accounts for non-sampling error [37]. The values of k_e3_ were typically only slightly larger than *k*_*e*_ (Additional file 1: Table S2), further suggesting that our sample sizes were sufficient to describe paternity in these species.

### Comparative analysis

To analyze how mean colony size parameters and nest site preference influence paternity frequency, paternity skew, and resulting intracolony genetic similarity (relatedness), we compared these traits across 22 vespine taxa, including 2 subspecies. Because species are not independent due to shared ancestry, we performed our analyses using methods accounting for phylogeny [43,44].

#### Data

We searched the literature for all vespine species with reports of paternity based on genetic data. We recorded the arithmetic mean number of patrilines per colony and used harmonic mean effective paternity to calculate intracolony genetic similarity (r). Where possible, we acquired colony-level paternity distribution data from authors to calculate the species mean B index of paternity skew for colonies with multiple paternity [38]. We then searched for data on average and maximum mature colony size (worker number). For species with multiple reports of colony size or effective mating frequency, we chose the study with the largest sample size, attempting to use reports of the two variables from the same population whenever possible, and (with the exception of *Vespa velutina*) only reports from colonies studied within the species’ native ranges. Our goal was to describe the average peak colony size of each species. Because colony sizes vary over the season, whenever possible we used averages of colonies that had adult reproductives present but had not entered decline (see Additional file 2). We similarly found data on the maximum number of workers recorded in an annual colony of each species, for colonies within the species’ native range. Finally, we also found estimates of the nest-site preferences of each species, recording the fraction of colonies with enclosed or subterranean nests (as opposed to aerial, exposed nests).

#### Phylogeny

We generated a phylogenetic hypothesis for our dataset using phyloGenerator [45] based largely on molecular data reported in two recent studies of vespine phylogeny [46,47] (Genbank accession numbers available in Additional file 1: Table S5). Sequences were aligned with MAFFT v6.847b [48], and the phylogeny was estimated with RAxML 7.3.0 [49] using a GTRGAMMA model and a single ML run with 1000 integrated bootstraps to determine support. We ultrametricized the resulting tree using the *chronopl* function in the R package *ape* with λ = 0 to approximate non-parametric rate smoothing [50]. Three species with trait data (*Vespula rufa, V. atropilosa,* and *D. norwegica*) were omitted from the analysis because no sequence data were available. Although our resulting tree closely matched the topology for *Vespula* and *Dolichovespula* of Lopez-Osorio et al. [47], the additional data used by Perrard et al. [46] led to a topology for *Vespa* that differed from ours. To ensure that this difference did not influence the outcome, we performed the same comparative analysis on a tree with a *Vespa* topology consistent with Perrard et al. [46] and all branch lengths set equal to 1.0 [51] (Additional file 1: Table S3, Additional file 1: Figure S1).

#### Statistics

Because species data are not independent due to shared history [43], we analyzed comparative data with phylogenetic generalized least squares (PGLS) models [52] implemented in R version 3.0.3 using the *caper* package [53,54]. To improve homoscedasticity we log-transformed colony size and inverse-transformed the skew index B. In each model we determined how well colony size and nest site predict intracolony genetic similarity (r), paternity frequency (k) or paternity skew (B), while accounting for phylogenetic history. We also performed a similar analysis using the maximum observed colony size rather than mean colony size for each species (Additional file 1: Table S4). In PGLS models, branch lengths of a phylogeny are used to correct for the non-independence of regression residuals, the basic assumption of ordinary least squares models that is violated by species data. The transformation parameter λ scales the branch lengths of the phylogeny and thus the degree to which the phylogeny affects the regression’s residuals. When λ = 0, the phylogeny is scaled to a star phylogeny and the analysis is equivalent to an ordinary least squares model. When λ = 1, PGLS is equivalent to Felsenstein’s independent contrasts [55], the most conservative analysis allowing the greatest effect of phylogeny. Using maximum likelihood methods, λ can be optimized to best fit the residuals of the regression model [56]. We ran each model twice, once with the maximum likelihood value of λ and, because the likelihood surfaces were often shallow, once with λ set to the upper 95% confidence interval value for the maximum likelihood estimate, for the most conservative analysis (i.e., allowing the greatest effect of phylogeny). To confirm that residuals were distributed normally, residual density plots were checked by eye.

## Results and discussion

The first aim of this study was to describe paternity in five species of *Vespula* wasps. The second aim was to determine if colony size and nest site predict intracolony genetic similarity (relatedness) across species, and if this pattern arises through effects of colony size and nest site on paternity frequency, paternity skew, or both. These patterns are used to address the question of why multiple mating and multiple paternity evolve.

### Paternity

All four species of the *Vespula rufa* group exhibited moderate multiple paternity, with most queens mating more than once, and some using the sperm from as many as 6 males (Additional file 1: Table S2). The estimated mean effective paternity values for species in the *Vespula rufa* group were moderate, varying between 1.5 and 2.1 (Table 1), and similar to *V. rufa*, which has a mean effective paternity of 1.5 [57]. Consequently, these species may provide interesting fodder for investigating the evolution of worker reproduction: when effective paternity is near 2.0, relatedness is not predicted to determine whether workers favor worker- or queen-derived males, as their relatedness to these two types of males is equal [58]. Thus, other factors such as costs of worker reproduction or the effectiveness of queen policing may usefully explain differences between these species. Behavioral observations suggest that such differences exist: some species appear to have frequent worker reproduction (e.g., *V. rufa* [57] and *V. consobrina* [59]), while others may have little or no worker reproduction in queenright colonies (*V. acadica* [60], *V. atropilosa* [61], and *V. vidua*, Chien and Loope, unpublished data).

The high average effective paternity of *Vespula flavopilosa* (~3.1) is similar to other members of the *Vespula vulgaris* group, though lower than other large-colony species in eastern North America [30]. This suggests no reversion to low paternity due to facultative social parasitism, as was also found in the similar social parasite *Vespula squamosa* [30]*.* This makes sense given that queens of both of these species eventually produce colonies with many workers, and thus likely benefit from greater paternity for the same reasons as other large-colony *Vespula.* It remains to be seen whether the *rufa*-group social parasite species (e.g., *V. infernalis* and *V. austriaca*), which lack a worker caste but are probably derived from a moderately polyandrous ancestor, have reverted to monandry as predicted by the hypothesis of benefits of a genetically diverse workforce [29,62].

### The effects of colony size and nest site on paternity frequency and paternity skew

**Figure 3:**
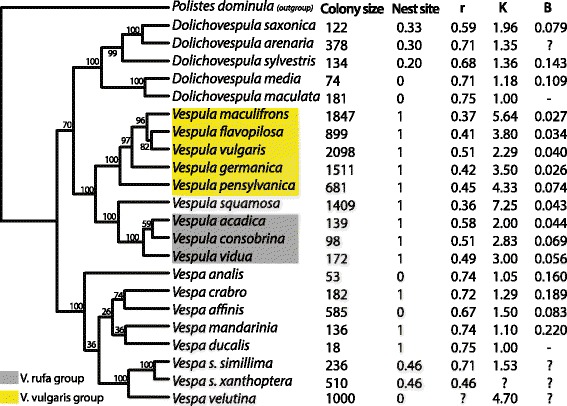
**Phylogeny and trait data used in comparative analyses.** The rate-smoothed phylogeny includes support values from 1000 integrated bootstraps in a single ML run using RAxML 7.3.0. Intracolony relatedness (*r*) was calculated from *k*_*e*_ or *k*_*e3*_. Paternity frequency (*k*) is the mean number of observed patrilines present in each colony. Colony size estimates are the arithmetic mean number of adult workers collected from mature colonies. Nest site values are the fraction of observed nests that are in enclosed sites (*ie* subterranean, tree and man-made structural cavities), as opposed to exposed, aerial nests. The *B* skew index was averaged across all multiple-paternity colonies. Data come from references [18,27,30,40,57,63-92]; for details see Additional file 2.

Our hypotheses for the evolution of multiple paternity all assume that ecology is largely responsible for colony size and nest site variation [21], and that paternity traits evolve in response to these two traits. To test whether colony size and nest site predict paternity traits, we conducted a phylogenetically controlled comparative analysis across 22 taxa (Figure 3). Our results suggest that (1) intracolony genetic diversity increases with colony size across species, and (2) large colony size is associated with both increased paternity frequency and reduced paternity skew (Table 2). We also confirm that paternity frequency and paternity skew explain much of the variation in intracolony genetic diversity, given that they, by definition, should together determine average intracolony relatedness.

**Table 2:** **PGLS models of the effect of colony size and nest site on paternity traits across 22 Vespine taxa**

**Model**	**Response**	**factors** ^**a**^	**λ** ^**b**^	**estimate**	***t***	***p***	**r** ^**c**^
**λ** **= ML**	Relatedness (*r*)	**log**_**10**_ **(** ***size*** **)** +	0.24^(na,0.83)^	−0.14	−3.89	**<0.001**	0.67^(0.3, 0.86)^
		***nest site***		−0.11	−2.27	**0.036**	0.46^(0.01, 0.76)^
	Paternity frequency (*k*)	**log**_**10**_ **(** ***size*** **)** +	0^(na,0.82)^	0.86	3.94	**<0.001**	0.67^(0.31, 0.86)^
		*nest site*		0.96	1.53	0.14	0.33^(−0.15, 0.68)^
	Paternity skew (*B* ^−1^)	**log**_**10**_ **(** ***size*** **)** +	0.35^(na,na)^	12.08	3.91	**<0.01**	0.72^(0.31, 0.91)^
		*nest site*		3.13	0.63	0.53	0.17^(−0.40, 0.64)^
	Relatedness (*r*)	***k*** **+**	1^(na,na)^	−0.04	−6.03	**<0.001**	0.85^(0.58, 0.95)^
		***B*** ^**−1**^		−0.004	−2.85	**0.014**	0.61^(0.11, 0.86)^
**λ** **= upper 95% CI**	Relatedness (*r*)	**log**_**10**_ **(** ***size*** **) +**	0.83	−0.13	−3.07	**0.007**	0.58^(0.16, 0.82)^
		*nest site*		−0.08	−1.30	0.211	0.29^(−0.20, 0.66)^
	Paternity frequency (*k*)	**log**_**10**_ **(** ***size*** **)** +	0.82	0.92	3.57	**0.002**	0.63^(0.25, 0.85)^
		*nest site*		0.57	0.66	0.51	0.15^(−0.33,0.57)^
	Paternity skew (*B* ^−1^)	log_10_(*size*)^d^ +	1	8.81	2.13	*0.052* ^d^	0.49^(−0.05, 0.81)^
		*nest site*		1.45	0.24	0.81	0.06^(−0.49, 0.58)^
	Relatedness (*r*)	***k*** **+**	1	−0.04	−6.03	**<0.001**	0.85^(0.58, 0.95)^
		***B*** ^**−1**^		−0.004	−2.85	**0.014**	0.61^(0.11, 0.86)^

^a^bold factors are significant at p<0.05.

^b^values in the λ = ML model show the maximum likelihood estimate of λ and the 95% confidence interval. “na” means the estimate is outside of the bounds (0, 1).

^c^effect size calculated from t-values and sample size using *compute.es* package in R [93]. Parenthetical values are 95% confidence intervals.

^d^effect of colony size on paternity skew is significant if nest site is omitted as a cofactor (*t* = 2.23, *p* = 0.042).

Our PGLS models simultaneously estimate regression coefficients and optimize the error structure of the residuals using the λ transformation [52,56,94], the recommended procedure for analyzing comparative data that may have phylogenetic signal. When λ is zero, the phylogenetic signal is estimated to be zero and the analysis is equivalent to ordinary least-squares regression, whereas when λ is set to 1, the model incorporates the maximum amount of phylogenetic covariance and is equivalent to independent contrasts analysis [55]. The maximum likelihood estimates of λ from our models are low to moderate, between zero and 0.35, for models including both colony size and nest site (Table 2), though the uncertainty in the λ estimates suggests a possibly large phylogenetic signal. Regardless, the most conservative analyses (models with λ set to the upper 95% confidence interval) yield similar significant effects of colony size on relatedness, paternity frequency, and paternity skew (Table 2). The alternative analyses using a different phylogeny (Additional file 1: Table S3) or maximum size as a proxy for colony size (Additional file 1: Table S4) give similar results. Unsurprisingly, actual variation in paternity skew and paternity frequency both significantly predict intracolony relatedness (Table 2). Overall, these results are consistent with the hypothesis that colony size influences intracolony relatedness through changes in paternity frequency and paternity skew. These findings for the Vespinae are in concord with previous analyses of more phylogenetically diverse sets of species [11,22].

**Figure 4:**
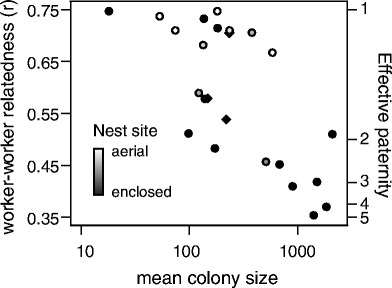
**Intracolony relatedness, effective paternity and colony size in Vespine wasps.** Each point represents mean trait values for a species (data shown in Figure 3 and Additional file 2). This figure includes the 21 species with relatedness and colony size data used in the analyses shown in Table 2, as well as three species (*Vespula atropilosa*, *V. rufa* and *Dolichovespula norwegica*) that were omitted due to lack of phylogenetic information (plotted as diamonds). Model results describing statistical relationships are reported in Table 2. Notably, *Vespa velutina* is not depicted due to lack of data on relatedness (only paternity frequency was reported), though this species has large mean colony size, high paternity frequency, and is an aerial nester.

We predicted that the nesting habit influences species’ paternity traits, as subterranean and cavity nesting may expose colonies to more pathogens. The higher paternities of the *rufa*-group *Vespula* species, compared with *Dolichovespula* species of similar colony size, suggest a role of nest site, as does the fact that most high paternity species (with the notable exception of *Vespa velutina*) are ground-nesting (Figure 4). Furthermore, when including colony size and nest site in a model predicting relatedness, both factors are significant when using the maximum likelihood value for λ in the PGLS model (Table 2). However, given the shallow likelihood surface for λ, the more conservative analysis with λ set to the upper 95% confidence interval suggests that colony size, but not nest site, drives the evolution of low intracolony relatedness. Nest site is also not a significant predictor of paternity frequency or paternity skew when included as a cofactor with colony size (Table 2). Nest site may be significant in the first model predicting relatedness (which is, because λ = 0, equivalent to an ordinary least squares model) because this trait is phylogenetically correlated with colony size and highly conserved in the genera *Vespula* and *Dolichovespula*. Although nest site is more variable in *Vespa*, it does not appear to be correlated with paternity in this group (analysis not shown). These mixed results provide little support for the hypothesis that pathogen-laden nest sites select for higher paternity, and the inclusion of additional species providing more phylogenetic contrasts (if they exist) would be useful.

### The evolution of multiple paternity in the Vespinae

We have considered the factors associated with the evolution of multiple paternity in a group of social wasps sharing similar annual life histories, colony founding strategies, natural enemies, temperate and subtropical distributions, foraging behaviors, and food sources [18,19,63,64]. Our results confirm an important role of large colony size in the evolution of high paternity in this group. This pattern is based partly on the correlated independent origins of large colony size and high paternity in the *vulgaris* and *squamosa* groups (or a single such origin and then correlated reduction of colony size and paternity in the *rufa* group; Figure 3). It is also supported by transitions to moderate colony size and moderate multiple mating in the hornets, by *Vespa simillima, Vespa velutina* and *Vespa affinis*, though these groups are in need of more study. Further evidence for a link between colony size and paternity would come from confirming the difference reported between subspecies of *V. simillima* [65]*,* and a detailed description of paternity and colony size for *V. velutina* in its native range.

What does the strong association with colony size tell us about the selective factors leading to multiple mating and multiple paternity in these species? The pathogens hypothesis is consistent with the observed effect of colony size, but remains to be directly tested in vespine wasps. The predictions that pathogens enter colonies via foragers, that larger colonies within species have more pathogens, and that species with larger colonies have more pathogens, are all testable. Numerous parasites and pathogens of social wasps have been identified [11,63,64] but studies of their relative occurrence and association with colony traits are lacking. The strongest evidence for this hypothesis come from experimental manipulation of mating frequency in bees [95,96]. Such an experiment would provide a powerful test of this hypothesis in vespine wasps.

The colony size prediction of the division of labor hypothesis rests on the existence of a difference between small- and large-colony species in behavioral organization. This could be the case if large-colony species possess more morphological or behavioral castes, improved division of labor based on genetically determined task thresholds, or a larger behavioral repertoire [16]. Such a pattern has been well documented in the polistine wasps, with greater specialization and evidence supporting a genetic basis underlying specialization, in large-colony species [97-99], as well as differences in task partitioning within species according to colony size [100]. However, there is little evidence for such a difference in the Vespinae [101]. Large-colony *Vespula* workers typically lack long-term specialization [102,103], and there is no evidence that they possess more complex, coordinated behaviors such as recruitment signals or task partitioning of nest construction [101]. On the other hand, studies of the division of labor and specialization in these wasps are few, and overlooked specialization or complexity in large-colony species, consistent with the division of labor hypothesis, may yet be discovered.

A third popular hypothesis, that queens require multiple mates to obtain enough sperm to last a lifetime [17,104], also predicts an association between multiple paternity and colony size. However, this hypothesis does not predict that queens of large-colony, highly polyandrous species use males’ sperm more evenly than their small-colony, slightly polyandrous counterparts (Table 2; [22]). Queens that are sperm limited should use all available sperm, and thus paternity skew should reflect the (presumably high) variation in male sperm availability. Therefore, our data suggest that selection for increasing genetic diversity, rather than selection to increase sperm quantity, may explain high paternity in the vespine wasps.

Additional hypothesized benefits of high paternity, such as reducing sex ratio conflict [6,7] or obtaining rare, critical patrilines [13,105,106], are not obviously linked to colony size, and thus seem less likely to explain multiple paternity in the vespine wasps. However, this group may provide useful subjects for further tests of these and other hypotheses, once more is known about their natural enemies, division of labor, mating biology, and sex investment. It will also be valuable to explore an alternative explanation for a link between colony size and paternity not considered here: it may be that increased paternity reduces intra-colony conflicts, increasing productivity and resulting in larger colonies [1,21,107].

## Conclusion

Our results show a strong association between colony size, paternity frequency and paternity skew in the vespine wasps, consistent with earlier, taxonomically broad, analyses. The observed patterns are consistent with hypotheses for the benefits of multiple paternity based on intracolony genetic diversity, but do not support the sperm limitation hypothesis. Clearly, further, more direct, tests of the pathogens hypothesis and the division of labor hypothesis are needed. Comparing closely related species with dramatically different paternity traits but otherwise similar natural history will help to reveal the details of when and why multiple paternity evolves.

## Availability of supporting data

In Additional file 2 we provide details of metadata and sources used for the comparative analysis. In Additional file 3 we provide the genotypes used in paternity assignments. In Additional file 4 we provide the sequences, alignments and tree from our phylogeny. Genbank accession numbers are found in (Additional file 1: Table S5).

## Additional files

Additional file 1:
**Table S1.** Allele number and diversity. **Table S2.** Colony data. **Table S3.** Alternative phylogeny model results. **Table S4.** Maximum colony size analysis. **Table S5.** Genbank accession numbers. **Figure S1.** Alternative phylogeny. Additional file 2:
**Data, sources and notes for comparative analysis.** This file includes a summary of comparative trait data with notes on sources. Additional file 3:
**Genotype data.** This file contains genotypes used in paternity estimates. Additional file 4:
**Phylogenetic data.** This file contains the sequences, alignments and tree from our phylogeny.
