# Supplementary material for: Colony size is linked to paternity frequency and paternity skew in yellowjacket wasps and hornets
Source: BMC Evol Biol. 2014 Dec 30;14:2625. doi: 10.1186/s12862-014-0277-x (PMC4298054; doi:10.1186/s12862-014-0277-x)
Supplement: Additional file 1: — Table S1. Allele number and diversity. Table S2. Colony data. Table S3. Alternative phylogeny model results. Table S4. Maximum colony size analysis. Table S5. Genbank accession numbers. Figure S1. Alternative phylogeny. [file 12862_2014_277_MOESM1_ESM.docx]

**Additional File 1: Supplemental Tables and Figures**

**Table S1:** Allele counts and diversity

**Table S2:** Descriptive colony data

**Table S3:** Alternative phylogeny model results

**Table S4:** Maximum colony size analysis

**Table S5:** Genbank accession numbers

**Figure S1:** Alternative phylogeny

**Table S1**. The number of alleles (above) and expected heterozygosity*^a^* (below) at each locus.

|  | List  2004 | List  2009 | | List  2019 | | Rufa  05 | Rufa  13 | Rufa  15 | Rufa  18 | Rufa  19 | VMA  3 | VMA  6 |
| --- | --- | --- | --- | --- | --- | --- | --- | --- | --- | --- | --- | --- |
| *V. acadica* | 5  0.66 | |  | |  | 9  0.83 |  | 13  0.87 | 12  0.86 |  |  | 5  0.52 |
| *V. atropilosa* |  | |  | |  | 5  0.68 | 10  0.81 | 12  0.74 | 11  0.84 |  |  |  |
| *V. consobrina* | 8  0.83 | |  | | 7  0.66 | 14  0.89 | 18  0.88 | 14  0.86 |  |  | 20  0.93 |  |
| *V. vidua* |  | | 3  0.5 | |  | 8  0.79 | 20  0.90 | 15  0.89 |  | 12  0.85 | 18  0.91 |  |
| *V. flavopilosa* | 5  0.55 | |  | |  | 5  0.67 | 5  0.69 |  |  | 9  0.77 | 17  0.87 |  |

*a*. Expected heterozygosity was calculated from adjusted allele frequencies determined by Colony2 after assigning parentage and accounting for family structure.

| **Colony** | ***n_w_*** | ***k*** | ***k_e_*** | ***k_e3_*** | ***B*** | **# W** | **Q?** |
| --- | --- | --- | --- | --- | --- | --- | --- |
| VAC1 | 19 | 1 | 1.00 | 1.00 | - | - | - |
| VAC2 | 20 | 1 | 1.00 | 1.00 | - | - | - |
| VAC3 | 20 | 2 | 2.00 | 2.10 | -0.025 | - | - |
| VAC4 | 20 | 3 | 2.47 | 2.65 | 0.0383 | - | - |
| VAC5 | 20 | 3 | 1.94 | 2.03 | 0.1483 | - | - |
| VAC6 | 20 | 3 | 2.06 | 2.17 | 0.1183 | - | - |
| VAC7 | 20 | 1 | 1.00 | 1.00 | - | - | - |
| VAC8 | 20 | 1 | 1.00 | 1.00 | - | - | - |
| VAC9 | 20 | 3 | 2.67 | 2.89 | 0.0083 | - | - |
| VAC10 | 20 | 2 | 2.00 | 2.10 | -0.025 | - | - |
|  |  |  |  |  |  |  |  |
| VAT1 | 20 | 2 | 2.00 | 2.10 | -0.025 | - | - |
| VAT2 | 20 | 4 | 2.60 | 2.81 | 0.0975 | - | - |
| VAT3 | 20 | 3 | 2.38 | 2.55 | 0.0533 | - | - |
| VAT4 | 19 | 1 | 1.00 | 1.00 | - | - | - |
| VAT5 | 23 | 2 | 1.91 | 1.98 | 0.002 | - | - |
| VAT6 | 20 | 2 | 1.72 | 1.79 | 0.055 | - | - |
| VAT7 | 20 | 2 | 1.10 | 1.11 | 0.38 | - | - |
| VAT8 | 20 | 4 | 2.47 | 2.65 | 0.117 | - | - |
| VAT9 | 20 | 2 | 1.34 | 1.36 | 0.22 | - | - |
| VAT10 | 19 | 2 | 1.87 | 1.95 | 0.008 | - | - |
|  |  |  |  |  |  |  |  |
| VC1 | 20 | 4 | 2.60 | 2.81 | 0.097 | 91 | Y^a^ |
| VC2 | 20 | 2 | 1.98 | 2.08 | -0.02 | 99 | Y |
| VC3 | 18 | 1 | 1.00 | 1.00 | - | 185 | Y |
| VC4 | 20 | 2 | 1.98 | 2.08 | -0.02 | 49 | Y |
| VC5 | 19 | 4 | 3.42 | 3.89 | -0.01 | 79 | N |
| VC6 | 20 | 4 | 3.23 | 3.60 | 0.022 | - | Y |

| **Colony** | ***n_w_*** | ***k*** | ***k_e_*** | ***k_e3_*** | ***B*** | **# W** | **Q?** |
| --- | --- | --- | --- | --- | --- | --- | --- |
| VC7 | 20 | 1 | 1.00 | 1.00 | - | 39 | Y^a^ |
| VC8 | 20 | 2 | 1.92 | 2.01 | -0.005 | 98 | Y |
| VC9 | 20 | 6 | 3.64 | 4.15 | 0.067 | 140 | Y |
| VC10 | 20 | 3 | 1.65 | 1.71 | 0.238 | 166 | Y^a^ |
| VC11 | 24 | 3 | 2.80 | 3.01 | -0.003 | 34 | Y^a^ |
| VC12 | 23 | 2 | 1.19 | 1.20 | 0.319 | 91 | Y |
|  |  |  |  |  |  |  |  |
| VV1 | 18 | 4 | 2.66 | 2.91 | 0.085 | 128 | Y |
| VV2 | 21 | 4 | 2.64 | 2.85 | 0.093 | 193 | Y |
| VV3 | 20 | 5 | 2.47 | 2.65 | 0.165 | 316 | Y |
| VV4 | 20 | 3 | 2.06 | 2.17 | 0.118 | 336 | Y |
| VV5 | 20 | 2 | 1.98 | 2.08 | -0.02 | 153 | Y |
| VV6 | 20 | 2 | 1.92 | 2.01 | -0.005 | 69 | Y^b^ |
| VV7 | 20 | 1 | 1.00 | 1.00 | - | 111 | Y |
| VV8 | 20 | 5 | 3.85 | 4.44 | 0.02 | 72 | Y |
| VV9 | 20 | 2 | 2.00 | 2.10 | -0.025 | - | - |
| VV10 | 19 | 2 | 1.86 | 1.94 | 0.071 | - | - |
|  |  |  |  |  |  |  |  |
| VF1 | 20 | 3 | 2.41 | 2.58 | 0.048 | - | - |
| VF2 | 20 | 4 | 2.67 | 2.89 | 0.087 | 1839 | Y |
| VF3 | 20 | 4 | 3.17 | 3.54 | 0.027 | 1755 | Y^a^ |
| VF4 | 20 | 3 | 1.94 | 2.03 | 0.148 | 214 | Y |
| VF5 | 20 | 3 | 2.41 | 2.58 | 0.048 | 1580 | Y |
| VF6 | 20 | 4 | 4.00 | 4.66 | 0.037 | 183 | Y |
| VF7 | 20 | 4 | 3.51 | 3.98 | 0.002 | 910 | Y |
| VF8 | 19 | 5 | 3.97 | 4.65 | 0.01 | 130 | Y^c^ |
| VF9 | 19 | 2 | 1.87 | 1.95 | 0.008 | 280 | Y |
| VF10 | 18 | 6 | 4.76 | 5.92 | 0.003 | 1199 | Y |

**Table S2:** Descriptive data for colonies of *Vespula acadica* (VAC), *V. atropilosa* (VAT), *V. consobrina* (VC), *V. vidua* (VV), *V. flavopilosa* (VF). *n_w_* is number of workers successfully genotyped, *k* is number of male mates detected, *k_e_* is an estimate of effective paternity, *k_e3_* is a corrected estimate of effective paternity (see Methods). *B* is paternity skew, # W is number of workers collected, and Q indicates presence of queen at collection. a. queen not present in sample but genotyped eggs/young larvae were diploid, suggesting colony likely queenright. b. queen collected but not genotyped. c. queen collected but desiccation suggests she had died before collection.

Alternative Analysis 1: Topology from Perrard et al. 2013 and Lopez-Osorio et al. 2014

**Table S3.** Comparative analyses of colony size and nest site on effective mating frequency across 21 species of Vespine wasps using alternative phylogeny based on topology of [46, 47]

| **Model** | **Response** | **Factors^a^** | **λ*^b^*** | **estimate** | ***t*** | ***p*** | **r*^c^*** |
| --- | --- | --- | --- | --- | --- | --- | --- |
| **λ=ML** | Relatedness (*r*) | **log_10_(*size*)** + | 0^(na,0.88)^ | -0.16 | -4.69 | **<0.001** | 0.73^(0.4, 0.89)^ |
|  |  | ***nest site*** |  | -0.12 | -2.60 | **0.018** | 0.51^(0.07, 0.79)^ |
|  | Paternity frequency (*k*) | **log_10_(*size*)** + | 0^(na,0.63)^ | 0.90 | 4.28 | **<0.001** | 0.70^(0.36, 0.88)^ |
|  |  | *nest site* |  | 1.01 | 1.65 | 0.12 | 0.35^(-0.12, 0.70)^ |
|  | Paternity skew (*B*^-1^) | **log_10_(*size*)** + | 0.42^(na,na)^ | 12.47 | 3.07 | **<0.01** | 0.63^(0.15, 0.87)^ |
|  |  | *nest site* |  | 0.94 | 0.20 | 0.84 | 0.05^(-0.49, 0.57)^ |
|  | Relatedness (*r*) | ***k* +** | 0^(na,na)^ | -0.05 | -6.41 | **<0.001** | 0.86^(0.61, 0.96)^ |
|  |  | ***B*^-1^** |  | -0.006 | -4.35 | **<0.001** | 0.76^(0.38, 0.92)^ |
| **λ= upper 95% CI** | Relatedness (*r*) | **log_10_(*size*) +** | 0.88 | -0.12 | -2.76 | **0.013** | 0.53^(0.10, 0.80)^ |
|  |  | *nest site* |  | -0.07 | -1.20 | 0.25 | 0.27^(-0.22, 0.64)^ |
|  | Paternity frequency (*k*) | **log_10_(*size*)** + | 0.63 | 0.89 | 3.60 | **0.002** | 0.64^(0.25, 0.85)^ |
|  |  | *nest site* |  | 0.73 | 0.94 | 0.36 | 0.21^(-0.27, 0.61)^ |
|  | Paternity skew (*B*^-1^) | **log_10_(*size*)** + | 1 | 10.32 | 2.27 | **0.040** | 0.52^(-0.02, 0.82)^ |
|  |  | *nest site* |  | -1.27 | -0.26 | 0.79 | 0.07^(-0.53, 0.66)^ |
|  | Relatedness (*r*) | ***k* +** | 1 | -0.04 | -6.48 | **0.000** | 0.87^(0.62, 0.96)^ |
|  |  | ***B*^-1^** |  | -0.003 | -2.21 | **0.045** | 0.51^(-0.03, 0.82)^ |

*a.* bold factors are significant at *p* < 0.05.

*b*. values in the λ=ML model show the maximum likelihood estimate for lambda and the 95% confidence interval. “na” means the estimate is outside of the bounds (0, 1).

*c.* Effect size was calculated from t-values and sample size using *compute.es* package in R. Parenthetical values are 95% confidence intervals.

**Figure S1.** Phylogeny used in analyses shown in Table 5.

Table S4: Comparative analyses of maximum colony size and nest site on effective mating frequency across 22 vespine taxa

| **Model** | **Response** | **factors^a^** | **λ*^b^*** | **estimate** | ***t*** | ***p*** | **r*^c^*** |
| --- | --- | --- | --- | --- | --- | --- | --- |
| **λ=ML** | Relatedness (*r*) | **log_10_(*size*)** + | 0.36^(na,0.93)^ | -0.11 | -2.49 | **0.023** | 0.64^(0.26, 0.85)^ |
|  |  | ***nest site*** |  | -0.06 | -0.90 | 0.11 | 0.37^(-0.11, 0.70)^ |
|  | Paternity frequency (*k*) | **log_10_(*size*)** + | 0^(na,0.91)^ | 0.94 | 3.90 | **0.001** | 0.67^(0.30, 0.86)^ |
|  |  | *nest site* |  | 0.79 | 1.22 | 0.24 | 0.27^(-0.21, 0.65)^ |
|  | Paternity skew (*B*^-1^) | **log_10_(*size*)** + | 0.26^(na,na)^ | 11.49 | 2.78 | **0.015** | 0.60^(0.09, 0.86)^ |
|  |  | *nest site* |  | 3.26 | 0.62 | 0.55 | 0.16^(-0.41, 0.64)^ |
|  | Relatedness (*r*) | ***k* +** | 1^(na,na)^ | -0.038 | -6.03 | **<0.001** | 0.85^(0.58, 0.95)^ |
|  |  | ***B*^-1^** |  | -0.004 | -2.85 | **0.014** | 0.61^(0.11, 0.86)^ |
| **λ= upper 95% CI** | Relatedness (*r*) | **log_10_(*size*) +** | 0.93 | -0.12 | -2.76 | **0.013** | 0.50^(0.05, 0.78)^ |
|  |  | *nest site* |  | -0.07 | -1.20 | 0.25 | 0.27^(-0.22, 0.64)^ |
|  | Paternity frequency (*k*) | **log_10_(*size*)** + | 0.91 | 0.99 | 3.71 | **0.002** | 0.65^(0.27, 0.85)^ |
|  |  | *nest site* |  | 0.20 | 0.22 | 0.82 | 0.05^(-0.44, 0.54)^ |
|  | Paternity skew (*B*^-1^) | **log_10_(*size*)** + | 1 | 7.12 | 1.76 | 0.10 | 0.43^(-0.14, 0.78)^ |
|  |  | *nest site* |  | 0.81 | 0.13 | 0.90 | 0.03^(-0.51, 0.56)^ |
|  | Relatedness (*r*) | ***k* +** | 1 | -0.038 | -6.03 | **<0.001** | 0.85^(0.58, 0.95)^ |
|  |  | ***B*^-1^** |  | -0.004 | -2.85 | **0.014** | 0.61^(0.11, 0.86)^ |

*a.* bold factors are significant at *p* < 0.05.

*b*. values in the λ=ML model show the maximum likelihood estimate for lambda and the 95% confidence interval. “na” means the estimate is outside of the bounds (0, 1).

*c.* Effect size was calculated from t-values and sample size using *compute.es* package in R. Parenthetical values are 95% confidence intervals.

|  | | **Gene** | | | | | | | | | |
| --- | --- | --- | --- | --- | --- | --- | --- | --- | --- | --- | --- |
| **Species** | **12S** | | **16S** | **28S** | **COI** | **H3** | **EF1a** | **COII** | **CytB** | **Apol2** | **win** |
| **Polistes dominula** | KJ147180.1 | | KJ147207.1 | KF981699.1 | KJ147236.1 |  | KF981673.1 | KJ147264.1 | KJ147292.1 |  | KF955646.1 |
| **Dolichovespula maculata** |  | | KJ147202.1 | KF981694.1 | KJ147231.1 |  | KF981668.1 | KJ147259.1 | KJ147287.1 | KF981643.1 | KF955641.1 |
| **Dolichovespula arenaria** | KJ147175.1 | | KJ147228.1 | KF981693.1 | KJ147230.1 |  | KF981667.1 | KJ147258.1 | KJ147286.1 | KF981642.1 | KF955640.1 |
| **Dolichovespula sylvestris** | KJ147179.1 | | KJ147206.1 | KF981698.1 | KJ147235.1 |  | KF981672.1 | KJ147263.1 | KJ147291.1 | KF981647.1 | KF955645.1 |
| **Dolichovespula media** | KJ147176.1 | | KJ147203.1 | KF981695.1 | KJ147232.1 |  | KF981669.1 | KJ147260.1 | KJ147288.1 | KF981644.1 | KF955642.1 |
| **Dolichovespula saxonica** | KJ147178.1 | | KJ147205.1 | KF981697.1 | KJ147234.1 |  | KF981671.1 | KJ147262.1 | KJ147290.1 | KF981646.1 | KF955644.1 |
| **Vespula vulgaris** |  | |  |  | GU207849.1 | |  |  |  |  |  |
| **Vespula pensylvanica** | KJ147197.1 | | KJ147224.1 | KF981715.1 | KJ147254.1 |  | KF981689.1 | KJ147282.1 | KJ147310.1 | KF981662.1 | KF955662.1 |
| **Vespula germanica** | KJ147194.1 | | KJ147221.1 | KF981712.1 | KJ147251.1 |  | KF981686.1 | KJ147279.1 | KJ147307.1 | KF981659.1 | KF955659.1 |
| **Vespula maculifrons** | KJ147196.1 | | KJ147223.1 | KF981714.1 | KJ147253.1 |  | KF981688.1 | KJ147281.1 | KJ147309.1 | KF981661.1 | KF955661.1 |
| **Vespula flavopilosa** | KJ147193.1 | | KJ147220.1 | KF981711.1 | KJ147250.1 |  | KF981685.1 | KJ147278.1 | KJ147306.1 | KF981658.1 | KF955658.1 |
| **Vespula squamosa** | KJ147198.1 | | KJ147225.1 | KF981716.1 | KJ147255.1 |  | KF981690.1 | KJ147283.1 | KJ147311.1 | KF981663.1 | KF955663.1 |
| **Vespula vidua** | KJ147199.1 | | KJ147226.1 | KF981717.1 | KJ147256.1 |  | KF981691.1 | KJ147284.1 | KJ147312.1 | KF981664.1 | KF955664.1 |
| **Vespula consobrina** | KJ147191.1 | | KJ147218.1 | KF981709.1 | KJ147248.1 |  | KF981684.1 | KJ147276.1 | KJ147304.1 | KF981657.1 | KF955657.1 |
| **Vespula acadica** | KJ147189.1 | | KJ147216.1 | KF981708.1 | KJ147246.1 |  | KF981682.1 | KJ147274.1 | KJ147302.1 | KF981655.1 | KF955655.1 |
| **Vespa crabro** | KJ147188.1 | | KJ147215.1 | KF981706.1 | KJ147244.1 |  | KF981680.1 | KJ147272.1 | KJ147300.1 | KF981653.1 | KF955653.1 |
| **Vespa affinis** | KJ147186.1 | | KJ147213.1 |  | KJ147242.1 |  | KF981678.1 | KJ147270.1 | KJ147298.1 |  | KF955651.1 |
| **Vespa ducalis** | KF933053.1 | | KF933064.1 | KF933076.1 | KF933084.1 | KF933092.1 |  |  |  |  |  |
| **Vespa simillima xanthoptera** | KF933049.1 | |  | KF933077.1 | KF933080.1 | KF933090.1 |  |  |  |  |  |
| **Vespa mandarinia** | KF933055.1 | | KF933062.1 | KF933068.1 | KF933085.1 |  |  |  |  |  |  |
| **Vespa analis** |  | | AB585940.1 |  | AB585948.1 |  |  | AB585957.1 |  |  |  |
| **Vespa simillima simillima** |  | |  |  | HM180937.1 | |  |  |  |  |  |
| **Vespa velutina** | KF933050.1 | | KF933058.1 | KF933073.1 | KF933081.1 | |  |  |  |  |  |

**Table S5:** Genbank accession numbers for sequences used in phylogenetic analysis
